# Sustainable Production of High-Performance Bioplastics from Agricultural and Industrial Biomass Waste by Integrating Deep Eutectic Solvent (DES) Pretreatment and Acetylation Processes

**DOI:** 10.1021/acsomega.4c09059

**Published:** 2025-03-11

**Authors:** Natcha Chyerochana, Quang Tam Huynh, Udomsap Jaitham, Paripok Phitsuwan, Kornkanok Aryusuk, Surat Hongsibsong, Ku-Fan Chen, Ken-Lin Chang

**Affiliations:** † Division of Biochemical Technology, School of Bioresources and Technology, King Mongkut’s University of Technology Thonburi, Bangkuntien, Bangkok 10140, Thailand; ‡ Institute of Environmental Engineering, National Sun Yat-Sen University, Kaohsiung 804, Taiwan; § School of Health Sciences Research, Research Institute for Health Sciences, Chiang Mai University, Chiang Mai 50200, Thailand; ∥ Environment, Occupational Health Sciences and Non-Communicable Disease Center of Excellence, Research Institute for Health Sciences, Chiang Mai University, Chiang Mai 50200, Thailand; ⊥ Department of Civil Engineering, National Chi Nan University, Nantou 545, Taiwan; # Net Zero Emissions and Resource Recycling Technology Research Center, National Sun Yat-Sen University, Kaohsiung 804, Taiwan; ∇ Department of Public Health, College of Health Sciences, Kaohsiung Medical University, Kaohsiung 807, Taiwan; ○ Center for Emerging Contaminants Research, National Sun Yat-Sen University, Kaohsiung 804, Taiwan

## Abstract

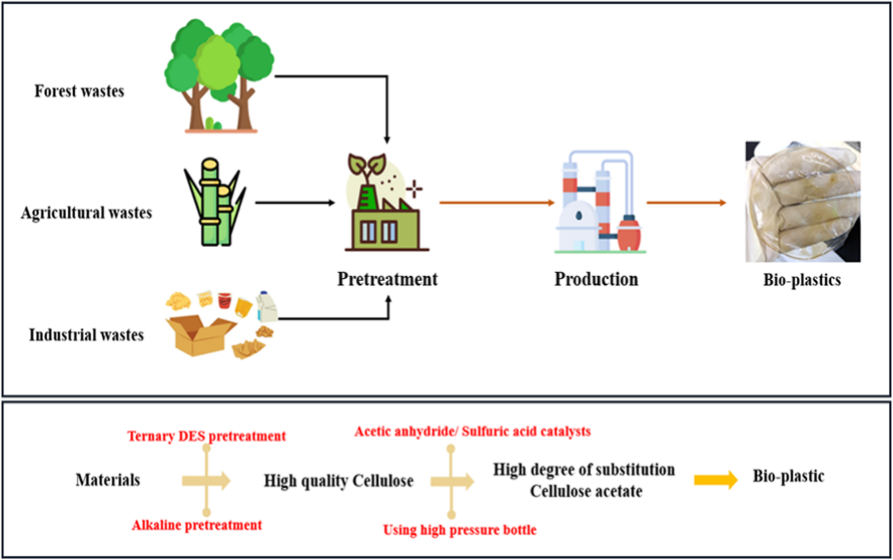

This study explores the production of bioplastic films from sugar cane bagasse, wood pulp waste, and boxboard waste using a three-step, sustainable process. First, cellulose was extracted from the biomass through a deep eutectic solvent (DES) pretreatment system composed of choline chloride, ethylene glycol, and oxalic acid (ChCl-EG-OA), which effectively removed lignin and enabled an efficient alkaline treatment for hemicellulose removal. Among the biomass sources, sugar cane bagasse yielded the highest cellulose content (72.86%), followed by wood pulp waste (43.82%) and boxboard waste (38.81%). In the second phase, optimal conditions for cellulose acetylation were established. Wood pulp waste achieved the highest cellulose acetate yield (81.25%), followed by boxboard waste (70.78%) and sugar cane bagasse (47.2%). Wood pulp waste-derived cellulose acetate also exhibited the highest acetyl content and degree of substitution (DS) at 2.83. In the final phase, bioplastic films derived from boxboard waste demonstrated superior mechanical properties, with a tensile strength of 11.23 MPa and elongation of 3.14%. In contrast, wood pulp waste-derived plastic exhibited moderate tensile strength (4.56 MPa) and minimal elongation (1.0%), while sugar cane bagasse-derived plastic showed the weakest performance. The study further highlights the adaptability of mixed-source bioplastics, as a blend of boxboard and wood pulp waste achieved a tensile strength of 7.26 MPa and elongation of 1.63%, illustrating the potential to enhance bioplastic properties through a biomass source combination. This approach contributes to the advancement of sustainable, high-performance bioplastics for a broad range of applications.

## 1 Introduction

Plastic products are highly expendable commodities in daily existence. Conventional plastics derived from petroleum, which have a low rate of decomposition, have emerged as a significant environmental concern.^1^ Landfills and oceans are particularly inundated with large quantities of disposable plastic, and the consequences of this pollution on wildlife, ecosystems, and human health are significant and detrimental.^2^ To solve this issue, it is necessary to develop inventive solutions that not only decrease the amount of plastic trash but also provide sustainable alternatives.

Biomass, an organic material derived from plants and animals, presents a promising avenue for creating sustainable materials that can replace traditional plastic materials. As a renewable resource, biomass can be processed into a variety of products, including biofuels, chemicals, and bioplastics.^3^ These bioplastics, unlike their petroleum-based counterparts, are often biodegradable and can significantly reduce the environmental footprint of plastic products.^2^ The utilization of biomass not only helps in managing waste but also supports the transition to a circular economy, where resources are reused and recycled.^4^ Among the various forms of biomass, cellulose stands out due to its abundance and versatility and can be converted into valuable products in different industries, such as paper, textile, electric, and biofuel.^5^ Cellulose is the most abundant organic compound on Earth. It is a straight-chain polymer without branching and is insoluble in water.^6^ The linear chain consists of glucose molecules connected by β-1,4-glycosidic connections. Specifically, two glucose molecules, known as cellobiose, form microfibrils through parallel alignment of crystalline structures using hydrogen bonds, hydrophobic contacts, and van der Waals forces.^7^ Plant tissue is the primary source of cellulose, in which cellulose contains different types of plants such as cotton (more than 90%), bagasse (35–45%), bamboo (40–55%), flax (70–80%), hemp (75–80%), jute (60–65%), kapok (70–75%), ramie (70–75%), straw (40–50%), and wood (40–50%).^8^

One of the most effective and sustainable methods for cellulose synthesis involves the use of deep eutectic solvents (DES).^9^ These solvents are valued for their low toxicity, biodegradability, and versatility, making them ideal for various applications in pharmaceuticals, biotechnology, materials science, and catalysis.^10^ To further enhance their functionality, ternary deep eutectic solvents (ternary DES) have been developed involving the combination of three different components. This ternary approach allows for the fine-tuning of properties such as viscosity, polarity, and thermal stability, thereby expanding their applicability.^11^ The ternary ChCl/SA (succinic acid)/EG type DES helps to improve the overall leaching efficiency for cobalt recycling from spent lithium-ion batteries.^12^ The ternary TMAC–glycerol–urea DES is more effective for the protein extraction process than binary TMAC–Urea DES.^13^ The evolution from binary to ternary DES highlights the continuous effort to optimize solvent properties for diverse industrial needs, promoting sustainable practices and green chemistry.

Cellulose acetate is an essential product obtained through the process of cellulose esterification.^14^ In a review study, Teixeira et al. pointed that cellulose acetate is a versatile chemical compound that finds use in several industries such as textiles, plastics, camera accessories, thermoplastic molding, magnetic tapes, photographic films, combs, electrical equipment, and telephones, and there are two types of cellulose acetate: cellulose diacetate, which has a degree of substitution (DS) between 2 and 2.5, and cellulose triacetate, which has a DS greater than 2.8.^15^ The qualities and solubility of cellulose acetate are determined by the acetyl concentration and degree of substitution (DS) of the material.^16^ Cellulose acetate is commonly derived from cellulose crystal, wood pulp, or agricultural waste materials such as cotton byproducts, sugar cane bagasse, banana byproducts, wheat, rice straw, and other substances that contain a substantial amount of cellulose.^15−17^ The production of cellulose acetate from waste materials is both ecologically friendly and commercially beneficial.

Although cellulose acetate is widely used in plastic production,^18^ it faces various challenges that result in it receiving less attention compared to other bioplastics like PLA (polylactic acid) or PHA (polyhydroxyalkanoates). One major challenge is the need for extensive purification of the raw material, which can be resource-intensive and costly.^19^ This highlights the need for methods that are both cost-efficient and environmentally friendly. Additionally, cellulose acetate plastic, itself, tends to be more brittle compared to other plastics,^20^ limiting its applications in products that require high impact resistance and flexibility. These factors contribute to its lower adoption, despite its sustainable and biodegradable nature. To address these issues, previous studies have explored combining cellulose acetate with other plastics or reinforcing agents such as nanocellulose to improve mechanical properties. While effective, these approaches increase costs and raise environmental concerns, making the process less sustainable. Developing more sustainable and efficient methods is crucial to enhancing cellulose acetate’s appeal in the marketplace.

In this study, a comprehensive method for cellulose acetate plastic synthesis from sugar cane bagasse, wood pulp waste, and boxboard waste was proposed, emphasizing the use of ternary deep eutectic solvents for improved cellulose extraction from waste materials. After the cellulose was extracted and converted into cellulose acetate, plastic films were produced. Initially, the films made from wood pulp waste and sugar cane bagasse-based cellulose acetate exhibited poor mechanical properties and were prone to breaking. To address this issue, cellulose acetate derived from boxboard waste was incorporated with the aim of enhancing the mechanical strength. The findings demonstrate how waste materials can be effectively converted to bioplastics with tailored mechanical properties, making them suitable for applications such as packaging. This innovative process leverages sustainable resources, avoids toxic chemicals, and promotes environmental safety and efficiency in producing high-quality plastic materials.

## 2 Materials and Methodology

### 2.1 Materials

Unless otherwise specified, all chemicals utilized in this study were commercially available and were not purified further. Acetic acid (C_2_H_4_O_2_, >99%), acetic anhydride (C_4_H_6_O_3_, 98%), and sulfuric acid (H_2_SO_4_, 98%) were purchased from Alfa Aesar (MA, USA). Choline chloride (ChCl, 99%), chloroform (CHCl_3_, 99%), and ethylene glycerol (C_2_H_6_O_3_, 99%) were purchased from Thermo Scientific (Belgium, China). Oxalic acid anhydrous (C_2_H_2_O_4_, 99%) and malonic acid (C_3_H_4_O_4_, 98%) were obtained from Guangdong Youhe Trading Co. (Taipei, Taiwan). Formic acid (CH_2_O_2_, 98%) was purchased from Sigma-Aldrich Chemistry (Darmstadt, Germany).

Boxboard waste and wood pulp waste were collected in the southern region of Taiwan. Sugar cane bagasse (SCB) originated in Thailand. About 500 g of biomasses was cut into minute fragments and desiccated at 105 °C until a constant weight was achieved. The samples were then ground and sieved through a 100 mesh screen in preparation for subsequent chemical and physical analyses. The characterization of sugar cane bagasse, boxboard waste, and wood pulp waste is presented in Table S1.

### 2.2 Synthesis of DESs

Deep eutectic solvents (DES) used in this study were referred to as ternary DES and were prepared by physical mixing of the constituent chemicals, following a method similar to that reported in previous research.^21^ A hydrogen bond acceptor (HBA) and two hydrogen bond donors (HBD), namely, choline chloride (ChCl) as the HBA and ethylene glycol (EG) as one of the HBDs were used. The fixed molar ratio was 1:2. For the experiment testing the effect of acid as the second HBD, oxalic acid, formic acid, and malonic acid were weighed using a standardized method and mixed in the correct molar ratios, as specified in Table S2. The resulting mixture was continuously stirred at 120 °C until a homogeneous, colorless liquid without any residual solids was obtained. Subsequently, the DESs were stored in a desiccator to prevent any further absorption of moisture until it was needed.

### 2.3 Extraction and Purification of Cellulose from Biomass

Initially, biomass and DES were mixed in a ratio of 1:20 (g/mL) in a hydrothermal autoclave reactor. The mixture was heated at 130 °C for 2 h. Post-treatment, an 80% ethanol/water solution (40 mL) was added and stirred at 200 rpm for 1 h further, followed by filtration to separate solid fractions (cellulose and hemicellulose) and liquid. The solid fraction was washed with deionized water and dried at 60 °C.

To obtain pure cellulose, we further treated the material to eliminate hemicellulose. This treatment involved mixing the biomass with a 5 wt % NaOH solution and a 30% H_2_O_2_ solution in a specific ratio: 1 g of biomass, 20 mL of NaOH solution, and 2 mL of H_2_O_2_ solution. The mixture was heated to 90 °C and maintained for 2 h. Following this chemical processing, the sample was filtered and thoroughly washed with distilled water to remove any remaining chemicals.

### 2.4 Acetylation of Cellulose Extraction

The synthesis of cellulose acetate was conducted using a conventional homogeneous process, where cellulose reacts with acetic anhydride in a medium of acetic acid with sulfuric acid acting as a catalyst. Initially, 2 g of the previously extracted cellulose was mixed with 20 mL of acetic acid to swell the cellulose fibers in a 100 mL high-pressure bottle. After 15 min, the fibers were activated by adding 0.3 mL of concentrated sulfuric acid in 10 mL of acetic acid, and this mixture was left for 30 min. Subsequently, 10 mL of acetic anhydride was slowly added, and the reaction mixture was allowed to react for 6-h duration. The reaction mixture was maintained at a constant temperature of 40 °C under magnetic stirring. The resulting viscous solution of cellulose acetate was filtered, and 500 mL of cold distilled water was added to the filtrate to terminate the reaction. This mixture was agitated for 1 h, and the precipitate formed was vacuum-filtered and washed thoroughly with water and ethanol to remove any excess acetic acid and acetic anhydride. The cellulose acetate yield was the ratio between the weight of the resulting cellulose acetate and the weight of the using cellulose in the acetylation process following eq 1:^16,22^
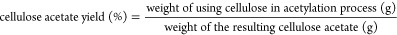
1

### 2.5 Degree of Substitution Measurement

Acetyl content and degree of substitution measurement (DS) were calculated following the study of Zakaria,^23^ Fei,^24^ and Homem.^25^ Dried cellulose acetate (0.5 g) was added to 50 mL of water, and the pH was adjusted to 7. Next, 25 mL of 0.5 N NaOH was added, and the mixture was heated with stirring until the precipitate dissolved and the solution became homogeneous. The solution was then allowed to cool. After it was cooled, the mixture was titrated with 0.5 N HCl until the pH returned to 7. The DS value was calculated using the titration formula provided. Blank titration with nonacetylated cellulose was also performed. The acetyl content and DS value were calculated using the titration eqs 2 and 3:
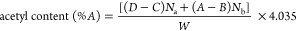
2
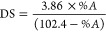
3where *A* and *B* are the volumes (mL) of NaOH solution required for the titration of the sample and blank, respectively; *C* and *D* are the volumes (mL) of HCl solution required for the titration of the sample and blank, respectively; *N*_a_ and *N*_b_ are the normalities of HCl solution and NaOH solution, respectively; and *W* is the mass (g) of the CA sample used.

### 2.6 Cellulose Acetate Plastic Film Production

2.5 g of the obtained cellulose acetate powder was dissolved in 3 mL of chloroform. The mixture was then combined with plasticizer and stirred for 60 min at 25 °C to ensure thorough incorporation of the additives and to enhance the homogeneity of the mixture. The resulting mixture was poured into a glass round frame and left to evaporate for 5 min. All of these steps were conducted in a cold room maintained at around 10 °C to slow the evaporation rate of the chloroform. Once the film dried, it was wetted with water, removed from the plate, and soaked in distilled water at 50 °C for 5 min to eliminate any residual solvent. To prepare the dried films for further analysis or testing, they were stored in a desiccator maintained at 25 °C and 50% relative humidity.

### 2.7 Component Analysis of Lignocellulosic Biomass

The measurement of the biomass component was conducted according to the guidelines provided by the National Renewable Energy Laboratory (NREL). Initially, 3 mL of sulfuric acid with a concentration of 72% was introduced into a high-pressure tube with a volume of 100 mL, along with 300 mg of the biomass sample. The mixture was then subjected to sonication for a duration of 1 h. For the second phase, 84 mL of water was introduced to the sample, which was subsequently inserted into an autoclave and subjected to a temperature of 120 °C for a duration of 1 h. The sample undergoes vacuum filtration, and the acid-insoluble component is rinsed with deionized water.

The quantity of lignin was determined using the following method: The acid-soluble lignin (ASL) concentration of the hydrolysates was measured by UV spectroscopy at a wavelength of 320 nm, utilizing a Shimadzu UV-1700 instrument from Kyoto, Japan. The acid-insoluble residue (AIR), which is the solid material left after filtration, was dried overnight at 105 °C and then subjected to a muffle furnace at 575 °C in order to measure the acid-insoluble ash (AIA). The Klason lignin (KL) content was determined by subtracting the acid-insoluble residue (AIR) from the acid-insoluble ash (AIA). The total lignin (TL) content was determined by adding together the ASL and KL values following eqs 4–6
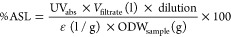
4
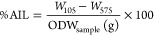
5where UV_abs_ is the average UV–vis absorbance at 320 nm. *V*_filtrate_ is the volume of filtrate, 86.73 mL. . ε: Absorptivity at 30 cm. ODW_sample_: Weight of biomass (g). *W*_105_: Weight of solid material at 105 °C. *W*_575_: Weight of solid material at 575 °C.
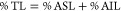
6

In order to determine the quantities of cellulose and hemicellulose, a 20 mL sample of the filtered liquid was extracted and then adjusted to a pH of 5–6 by neutralizing it with calcium carbonate. The sample was subsequently examined using high-performance liquid chromatography (HPLC) with a Hitachi CM5000 instrument from Japan. The analysis employed a BP-8000 Ca column measuring 300 × 7.8 mm, along with a refractive index (RI) detector. The mobile phase consisted of purified water flowing at a rate of 0.6 mL/min, while the column temperature was maintained at 85 °C. The analysis lasted 20 min. The percentage of cellulose or hemicellulose was obtained using eq 7:
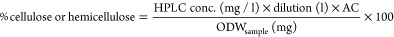
7where HPLC conc.: concentration of sugar as determined by HPLC, mg/mL. AC: Using an anhydrous correction of 0.88 for C-5 sugars and a correction of 0.90 for C-6 sugars. ODW_sample_: Weight of sample (mg).

### 2.8 Fourier Transform Infrared (FT-IR) Spectrophotometry

FT-IR technique was utilized to determine the functional groups present in cellulose and cellulose acetate, using a Thermo Nicolet iS5 Fourier transform infrared spectrometer from Thermo Fisher Scientific. This spectrometer operated with OMNIC software. The FT-IR spectrometer’s wavenumber range spans from 400 to 4000 cm^–1^ with an absorbance resolution of 0.9 cm^–1^ and wavenumber precision of 0.001 cm^–1^ at 2000 cm^–1^.

### 2.9 Moisture Content, Water Absorption, Biodegradability of Bioplastic Film

The technique of Sanyang,^26^ Vasile,^27^ and Rumi^28^ was modified to measure the moisture content, water absorption, and biodegradability of bioplastic sheets.

In order to find out how much water was in the Bioplastic, 1.5 cm^2^ samples were weighed to get their original weight (*w*_1_). After being put in an oven set to 50 °C for 24 h, these samples were dried. After the samples were dried, they were weighed again to obtain their final weight (*w*_2_). After that, eq 8 was used to figure out the moisture content:
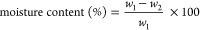
8

A slightly changed version of ASTM D570-98 was used to determine how much water the bioplastics could hold. Bioplastic pieces 1.5 cm^2^ were first dried in an oven at 50 °C for 24 h to find out their dry weight (*w*_1_). After being dried, these samples were put in a jar with 50 mL of distilled water and left there for 24 h at room temperature. After the time of soaking, the bioplastic samples were taken out of the water, and the extra water was filtered out. The pieces of bioplastic were weighed again after they had absorbed water to obtain their final weight (*w*_2_). Equation 9 was used to figure out how much water the bioplastics could hold:
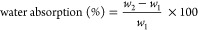
9

For biodegradability, 3.5-cm-wide pieces of bioplastic were first weighed to find their starting weight (*w*_1_). Once that was done, the samples were buried under 2 cm of wet garden dirt in Styrofoam cups. They stayed there for 5 days at room temperature. There was no change in the soil’s moisture level over the 5 days. The bioplastic residues were carefully taken from the soil after the required incubation time. After being washed with water, these leftovers were dried in an oven set to 50 °C for 24 h. The end weight (*w*_2_) of the bioplastic residues was measured after they had dried. Equation 10 was then used to figure out how biodegradable the bioplastic samples were:
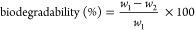
10

### 2.10 Measurement of Tensile Strength and Elongation

A digital force measurement (Isf-df100, China) was used to calculate the breaking force, and the cross-sectional area (mm^2^) was calculated by the width (mm) and thickness (mm) of the tested sample. Tensile strength (MPa) was identified by dividing the maximum force (kg) before fracture by the cross-sectional area, and the elongation (%) was calculated as the change in length (mm) at fracture divided by the original length (mm).

## 3 Result and Discussion

### 3.1 Cellulose Production

In this study, various biomasses, including sugar cane bagasse, wood pulp waste, and boxboard waste, were used for cellulose acetate synthesis. Among these sources, sugar cane bagasse is notable for its cellulose content of 37.77 ± 0.38%, hemicellulose level of 24.26 ± 1.08%, and lignin content of 23.70 ± 0.40%. This composition has a more intricate arrangement in contrast to boxboard waste and wood pulp waste, which possess lower proportions of hemicellulose constituents, as mentioned in Table S1. The intricate composition of sugar cane bagasse was initially chosen for cellulose production in this investigation with optimizing the conditions for cellulose synthesis. Once the optimal conditions are determined, the process will be expanded to include boxboard waste and wood pulp waste.

#### 3.1.1 The Effect of DESs Types on the Cellulose Production

In this experiment, various DESs with a molar ratio of HBA:HBD-1:HBD-2 of 1:2:0.6, which were selected based on findings from prior preliminary experiments, were employed to determine the most effective DES for the pretreatment process of SCB. Initially, 2 g of SCB was mixed with 20 mL of DES and subjected to a high-pressure autoclave at 130 °C for 2 h to facilitate lignin removal. Following the pretreatment, deionized (DI) water was used to dilute the DES and obtain the residue. This residue was then treated with 20 mL of 5% NaOH and 2 mL of H_2_O_2_ to remove hemicellulose and enhance the purity of the cellulose.

**Figure 1:**
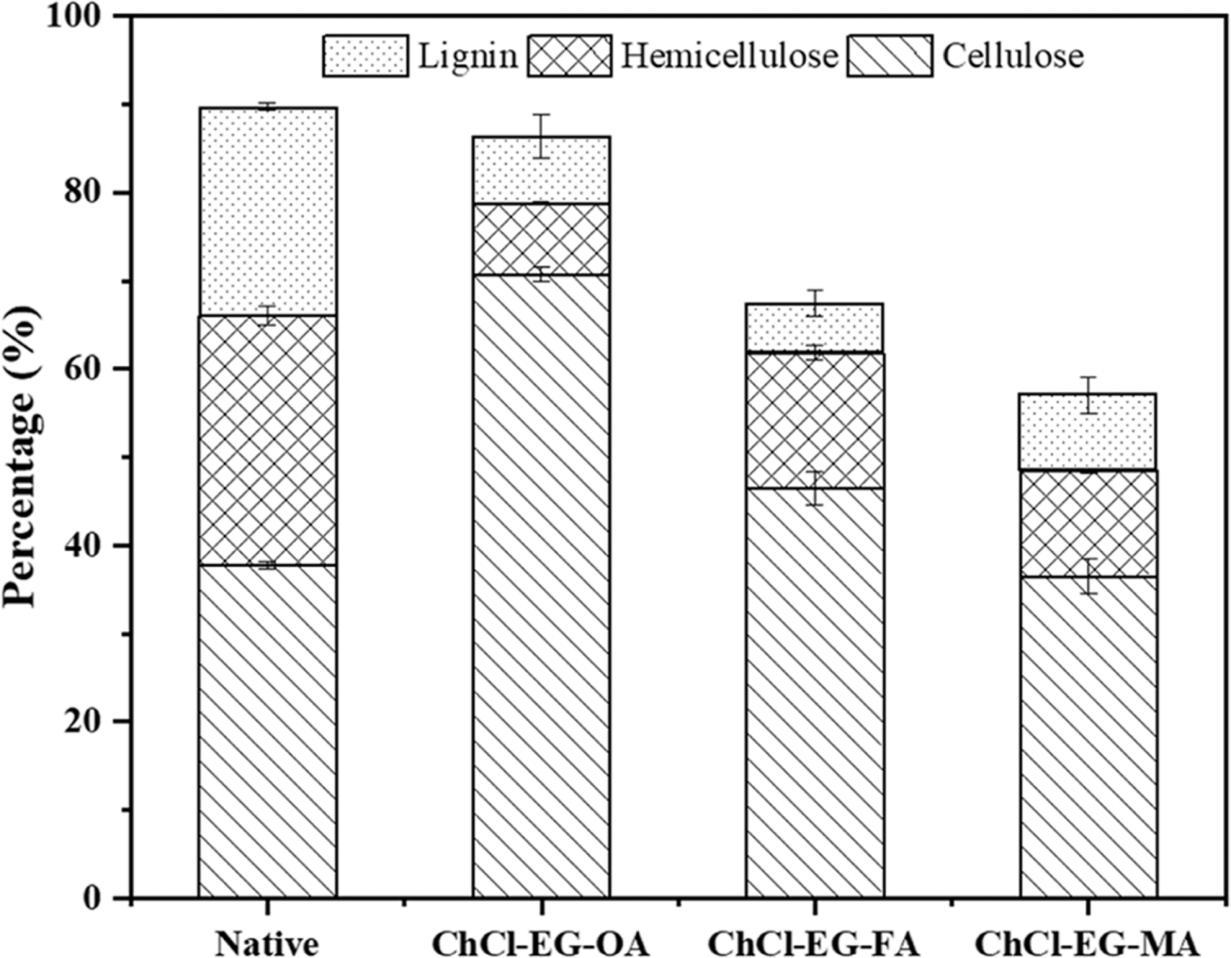
Effect of DESs types on cellulose production.

The final product obtained was a yellowish pulp, with the highest cellulose content (70.72%) achieved using ChCl-EG-OA. This was followed by ChCl-EG-FA with a cellulose content of 46.52% and ChCl-EG-MA with a cellulose content of 36.52%. The detailed results are displayed in Figure 1. The results indicate that ChCl-EG-OA was the most effective DES in increasing the cellulose content and reducing the amount of hemicellulose and lignin. The cellulose content in the treated SCB with ChCl-EG-OA was significantly higher compared with the native sample, which had a cellulose content of 37.77%. This suggests that ChCl-EG-OA has a superior capability to break down lignin and hemicellulose, thereby enhancing the cellulose concentration. Oxalic acid acts as a bidentate chelating agent,^29^ possessing two oxygen atoms with lone pairs of electrons that can form coordinate covalent bonds with metal ions such as calcium, magnesium, and iron, which are naturally present in biomass. These metals are often associated with lignin and hemicellulose. By binding to these metal ions, the chelating ability of oxalic acid can weaken the interactions between the metal ions and lignin or hemicellulose, thereby contributing to their breakdown during the biomass pretreatment process.

In contrast, formic acid, although a strong acid, lacks the chelating properties of oxalic acid, making it less effective at dissolving lignin. Malic acid, being a weaker acid, provides even less hydrolytic activity, resulting in lower cellulose purity. Specifically, ChCl-EG-FA-treated SCB showed a cellulose content of 46.52% and a lignin content of 5.56%, while ChCl-EG-MA-treated SCB had a cellulose content of 36.52% and a lignin content of 8.56%.

Furthermore, the hydrogen bond interactions between ChCl (acting as an HBA) and EG (acting as an HBD) create a highly polar environment^30^ that disrupts the hydrogen-bonding network in lignin and hemicellulose. This polar environment solubilizes these components effectively, while preserving the crystalline structure of cellulose. Together, these mechanisms explain the effectiveness of DES in biomass pretreatment.

#### 3.1.2 Effect of Oxalic Acid Ratio in ChCl-EG-OA DES on the Cellulose Production

This experiment identified the optimal molar ratio of OA in the ChCl-EG-OA mixture for the pretreatment of SCB. Various molar ratios of OA, ranging from 0.4 to 1, were evaluated. The reaction conditions involved mixing 2 g of biomass with 20 mL of DES and subjecting the mixture to a high-pressure autoclave at 130 °C for 2 h to primarily facilitate lignin removal. After DES pretreatment, DI was used to remove the DES and obtain the residue, which was then treated with 20 mL of 5% NaOH and 2 mL of H_2_O_2_ to remove hemicellulose.

**Figure 2:**
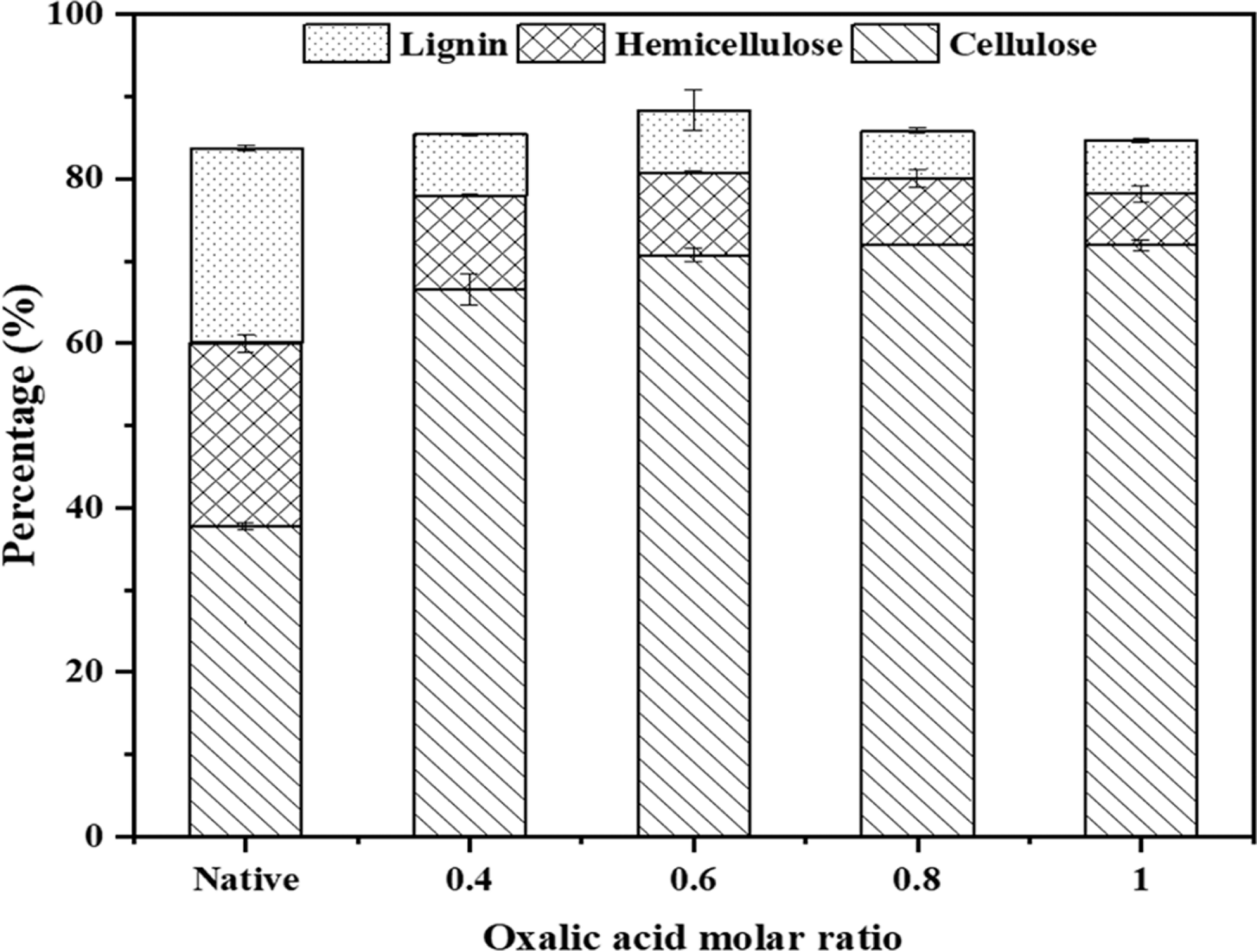
Ratio of oxalic acid in ChCl-EG-OA DES affects the synthesis of cellulose.

The results in Figure 2 showed a significant increase in cellulose content across all treated samples compared to the native sample (37.77%). The highest cellulose content was achieved at an oxalic acid molar ratio of 1, reaching 71.94%. This suggests that higher concentrations of oxalic acid are highly effective in breaking down lignin, thereby enriching the cellulose content. Hemicellulose content decreased in the treated samples, with the lowest content (8.12%) observed at an oxalic acid molar ratio of 0.8, indicating efficient removal by the subsequent NaOH treatment. Additionally, there was a significant reduction in lignin content across all treated samples, with the most substantial lignin removal (5.78%) at an oxalic acid molar ratio of 0.8.

Analyzing these findings, it is evident that an oxalic acid molar ratio of 0.8 provides a balanced and effective pretreatment, resulting in high cellulose content, significant lignin removal, and the lowest hemicellulose content. The increasing ratio of oxalic acid in the DES not only enhances lignin breakdown but also facilitates the subsequent NaOH treatment by making hemicellulose more accessible for removal.

#### 3.1.3 The Effect of Reaction Time, Reaction Temperature, and NaOH Concentration on the Alkaline Hydrolysis

To reduce the cost of time and chemicals, this study employed the response surface method (RSM) by using a Box–Behnken design. It utilized three variables: the reaction time, reaction temperature, and NaOH concentration. In total, 17 experiments were conducted to evaluate the correlation between the process parameters and the optimal generation of the cellulose extraction yield and elimination of lignin and hemicellulose from biomass. The content percentages of cellulose, hemicellulose, and lignin were chosen as the response. The respective variable values are tabulated in Table 1. To avoid systematic errors in the variables, the trials were conducted randomly, and each experiment was repeated three times per condition.

**Table 1:** Experimental Factors and Levels Used in Box–Behnken Design

	units	code	level		
independent variable units code			–1	0	1
pretreatment time	min	A	30	135	240
pretreatment temperature	°C	B	90	120	150
NaOH concentration	M	C	1	5.5	10

The experimental design results on the different reaction conditions are shown in Table 2. The results were analyzed using multiple regression analysis functions of the RSM methodology. A second-order polynomial equation was created by using the coded numbers to show the quantitative connection between the test variables and the biomass compounds.
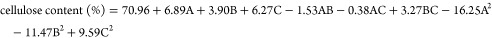
11
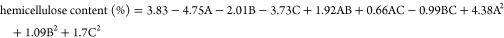
12
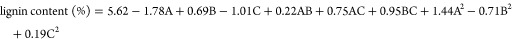
13where A, B, and C are the test variables of solution addition, treatment time, and pH level, respectively.

**Table 2:** Actual Experiment Data

entry	pretreatment time, min	pretreatment temperature, °C	NaOH concentration, M	cellulose content, %	hemicellulose content, %	lignin content, %
1	30(−1)	150(+1)	5.5(0)	41.23 ± 0.86	11.12 ± 0.96	9.13 ± 0.62
2	135(0)	150(+1)	10(+1)	68.23 ± 1.73	1.23 ± 0.22	5.32 ± 2.21
3	135(0)	150(+1)	1(−1)	41.12 ± 1.71	9.35 ± 1.25	6.17 ± 0.69
4	240(+1)	150(+1)	5.5(0)	51.3 ± 1.78	2.31 ± 1.03	5.01 ± 0.59
5	135(0)	90(−1)	10(+1)	52.13 ± 1.06	6.06 ± 0.48	2.1 ± 0.68
6	240(+1)	90(−1)	5.5(0)	48.32 ± 0.81	3.65 ± 1.93	3.12 ± 1.92
7	35(0)	90(−1)	1(−1)	38.12 ± 2.19	10.23 ± 1.4	6.78 ± 1.8
8	135(0)	120(0)	5.5(0)	72.86 ± 1.64	3.45 ± 0.73	6.01 ± 0.97
9	30(−1)	120(0)	1(−1)	35.23 ± 0.22	18.23 ± 0.48	9.91 ± 1.22
10	240(+1)	120(0)	1(−1)	50.45 ± 0.36	10.56 ± 1.79	5.86 ± 1.45
11	135(0)	120(0)	5.5(0)	69.85 ± 1.19	4.01 ± 0.22	6.23 ± 1.07
12	30(−1)	120(0)	10(+1)	40.56 ± 0.49	8.12 ± 1.9	7.12 ± 0.51
13	135(0)	120(0)	5.5(0)	73.2 ± 0.22	4.56 ± 1.65	5.1 ± 1.05
14	30(−1)	90(−1)	5.5(0)	32.1 ± 0.95	20.12 ± 1.29	8.13 ± 0.41
15	135(0)	120(0)	5.5(0)	69.56 ± 1.94	3.01 ± 1.83	5.12 ± 0.77
16	240(+1)	120(0)	10(+1)	54.23 ± 0.45	3.1 ± 0.92	6.1 ± 1.56
17	135(0)	120(0)	5.5(0)	69.36 ± 1.32	4.1 ± 0.68	5.63 ± 1.94

Based on the experimental data provided from the Box–Behnken design, the results in terms of ANOVA of the content of biomass compounds after the pretreatment are as follows.

The statistical analyses of the content percentage of cellulose, hemicellulose, and lignin reveal the significance of the models in predicting outcomes. For cellulose content (Table 3), the model is highly coefficient of determination (*R*^2^ = 0.9547). Similarly, the hemicellulose content model (Table 4) also shows a high *R*^2^ value of 0.9427, while *R*^2^ of the lignin content model (Table 5) at 0.9274, demonstrating that all models are effective in explaining the variability in their respective data sets.

**Table 3:** Values of the Regression Coefficients and the Analysis of Variance from Cellulose Content Percentage Analysis

source	sum of squares	df	mean square	*F*-value	*p*-value
model	3150.03	9	350	16.4	0.0006
A – time	380.6	1	380.6	17.83	0.0039
B – temperature	121.76	1	121.76	5.7	0.0483
C – NaOH	315.38	1	315.38	14.78	0.0063
AB	9.46	1	9.46	0.443	0.527
AC	0.6006	1	0.6006	0.0281	0.8715
BC	42.9	1	42.9	2.01	0.1992
A^2^	1112.59	1	1112.59	52.13	0.0002
B^2^	554.23	1	554.23	25.97	0.0014
C^2^	387.48	1	387.48	18.15	0.0037
residual	149.41	7	21.34		
lack of fit	135.03	3	45.01	12.52	0.0168
pure error	14.38	4	3.59		
cor total	3299.44	16			
*R*^2^	0.9547				
adjusted *R*^2^	0.8965				
predicted *R*^2^	0.3384				
adeq precision	11.3069				

**Table 4:** Values of the Regression Coefficients and the Analysis of Variance from Hemicellulose Content Percentage Analysis

source	sum of squares	df	mean square	*F*-value	*p*-value
model	451.06	9	50.12	12.79	0.0014
A – time	180.22	1	180.22	45.99	0.0003
B – temperature	32.2	1	32.2	8.22	0.0241
C – NaOH	111.45	1	111.45	28.44	0.0011
AB	14.67	1	14.67	3.74	0.0943
AC	1.76	1	1.76	0.448	0.5247
BC	3.9	1	3.9	0.9953	0.3517
A^2^	80.76	1	80.76	20.61	0.0027
B^2^	5.04	1	5.04	1.29	0.2939
C^2^	13.6	1	13.6	3.47	0.1048
residual	27.43	7	3.92		
lack of fit	25.98	3	8.66	23.81	0.0052
pure error	1.45	4	0.3637		
cor total	478.49	16			
*R*^2^	0.9427				
adjusted *R*^2^	0.869				
predicted *R*^2^	0.1266				
adeq precision	12.6143				

**Table 5:** Values of the Regression Coefficients and the Analysis of Variance from Lignin Content Percentage Analysis

source	sum of squares	df	mean square	*F*-value	*p*-value
model	54	9	6	9.94	0.0031
A – time	25.21	1	25.21	41.76	0.0003
B – temperature	3.78	1	3.78	6.26	0.0408
C – NaOH	8.16	1	8.16	13.52	0.0079
AB	0.198	1	0.198	0.3281	0.5847
AC	2.3	1	2.3	3.8	0.0922
BC	3.67	1	3.67	6.08	0.0432
A^2^	8.76	1	8.76	14.51	0.0066
B^2^	2.14	1	2.14	3.54	0.1018
C^2^	0.1476	1	0.1476	0.2446	0.636
residual	4.22	7	0.6036		
lack of fit	3.18	3	1.06	4.06	0.1047
pure error	1.04	4	0.2612		
cor total	58.22	16			
*R*^2^	0.9274				
adjusted *R*^2^	0.8341				
predicted *R*^2^	0.098				
adeq precision	14.0177				

In terms of individual factors, time, temperature, and NaOH concentration showed high significance across all models. Overall, the trend across the three models shows that the most significant factor is pretreatment time (*p* = 0.0039 for cellulose and 0.0003 for hemicellulose and lignin), followed by NaOH concentration (*p* = 0.0063 for cellulose, *p* = 0.0011 for hemicellulose, and *p* = 0.0079 for lignin) and pretreatment temperature (*p* = 0.0483 for cellulose, *p* = 0.0241 for hemicellulose, and *p* = 0.0408 for lignin). Furthermore, the quadratic terms are also significant in most cases, indicating that these factors have nonlinear effects on the content percentages. The interaction terms, however, are generally not significant, except for some specific interactions in the lignin model, suggesting that the combined effects of these factors are less influential.

The predicted *R*^2^ values for cellulose (0.3384), hemicellulose (0.1266), and lignin (0.0980) are considerably lower than their adjusted *R*^2^ with cellulose at 0.8965, hemicellulose at 0.8690, and lignin at 0.8341, indicating a better explanatory power than predictive power. The adequate precision values, which provide a signal-to-noise ratio, are high for cellulose (11.3069), hemicellulose (12.6143), and lignin (14.0177), suggesting that all models are reliable and effectively explain the observed trends.

**Figure 3:**
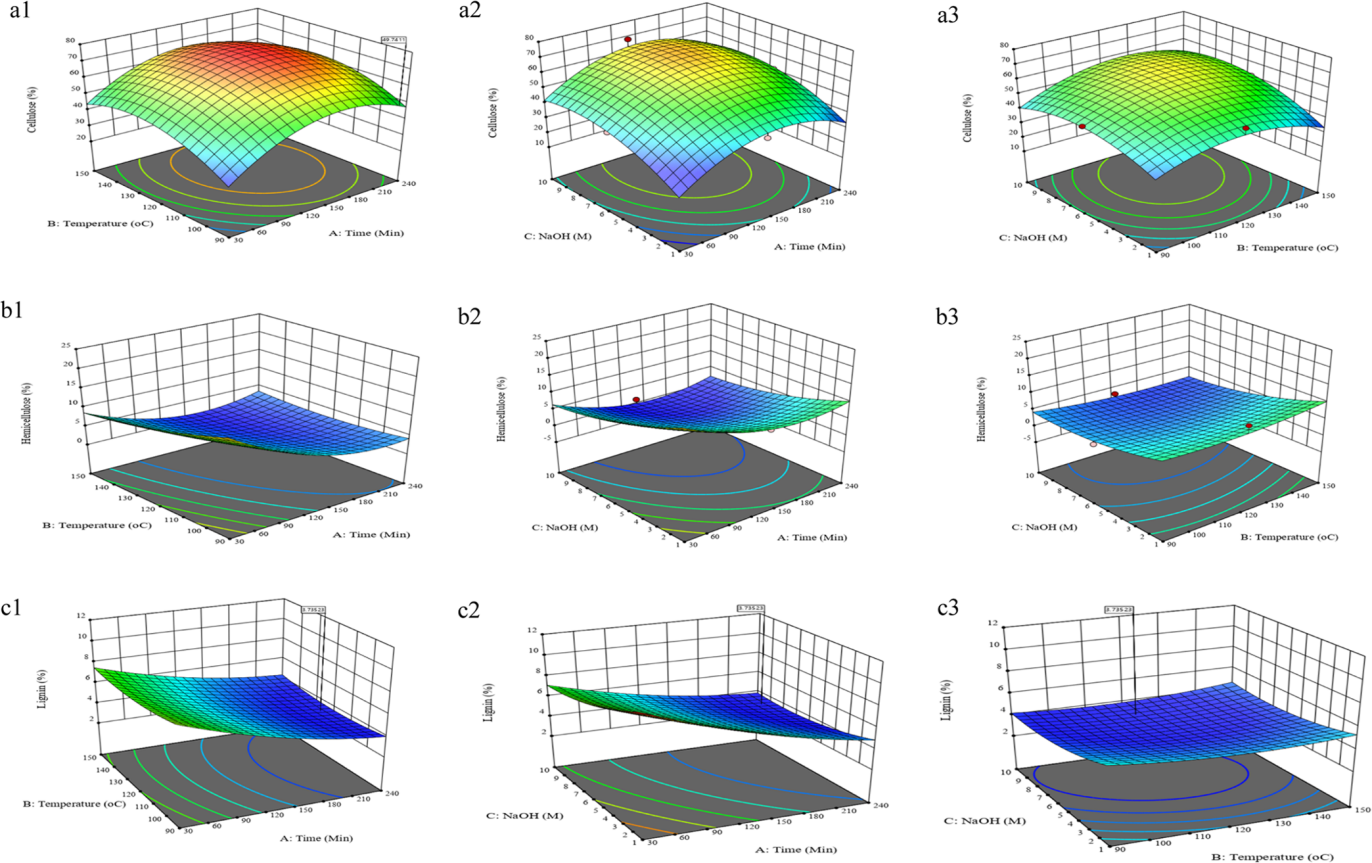
Response surface (3D) and contour (2D) plots of reaction parameter effects on (a) cellulose, (b) hemicellulose, and (c) lignin content (1, treatment time and temperature; 2, treatment time and NaOH concentration; 3, NaOH concentration and temperature).

The response surface (3D) and contour (2D) plots of reaction parameter effects on the cellulose, hemicellulose, and lignin contents are displayed in Figure 3. The surface plots for cellulose content (Figure 3a) indicate that the highest cellulose content peaks present at temperatures between 120 and 130 °C, with a pretreatment time of 120–150 min and NaOH concentrations ranging from 5 to 10 M. These conditions suggest that a moderate duration, moderately high temperature, and high NaOH concentration are optimal for maximizing cellulose extraction. In contrast, the surface plots for hemicellulose (Figure 3b) and lignin (Figure 3c) content reveal the conditions under which their levels are minimized. Hemicellulose content is lowest at higher temperatures (above 120 °C) and only shorter pretreatment times (30–90 min), particularly with lower NaOH concentrations (1 to 5 M). For instance, the lowest hemicellulose content (1.23%) was achieved at 135 min, 150 °C, and 10 M NaOH. Similarly, lignin content is minimized at temperatures between 120 and 150 °C, shorter pretreatment times, and NaOH concentrations around 5.5 M. The lowest lignin content (2.1%) was found at 240 min, 150 °C, and 5.5 M NaOH. Importantly, the lignin content results indicate that a pretreatment time of 90 min is sufficient, as extending the time to 240 min does not significantly reduce lignin further. This finding aligns with and emphasizes the effectiveness of the prior DES pretreatment, which has been shown to be effective in lignin removal.

The combination of DES and NaOH pretreatment significantly reduced the duration and severity of the subsequent alkaline process, enabling the use of lower NaOH concentrations. This synergy minimizes chemical consumption, operational costs, and waste generation, enhancing the overall efficiency and sustainability of the process. By combining these methods, a practical approach to cellulose purification was achieved.

Following the results of RSM, the optimal condition at temperatures of 120 °C, with a pretreatment time of 135 min and NaOH concentrations ranging from 5.5 M, was chosen to employ for wood pulp waste and boxboard waste.

**Figure 4:**
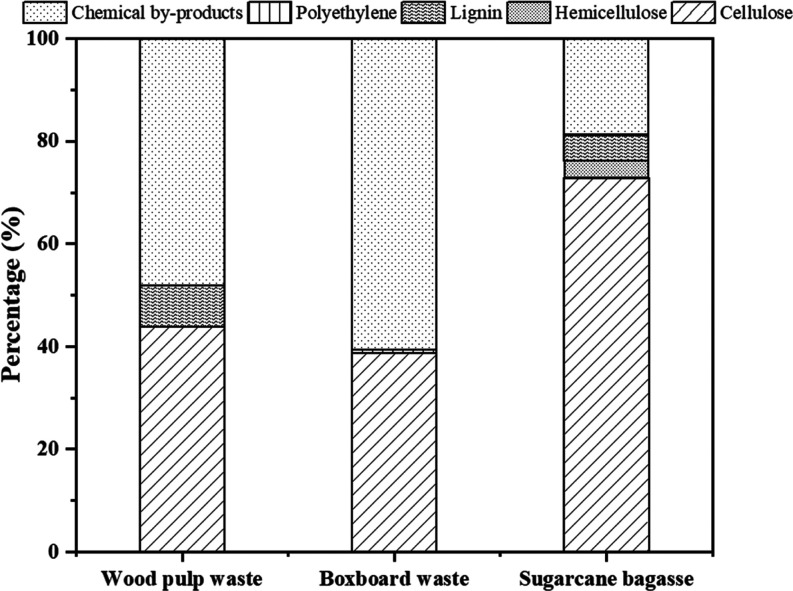
Effect of the treatment method on various types of biomass.

As displayed in Figure 4, sugar cane bagasse shows the highest cellulose content at 72.86%, along with 3.45% hemicellulose and 5.01% lignin. Wood pulp waste has a substantial cellulose content of 43.82% but also contains 8.16% lignin, which might influence further processing. Boxboard waste, although free of hemicellulose and lignin post-pretreatment, has a lower cellulose content at 38.81%. In addition, the composition of boxboard waste contains the presence of 0.59% polyethylene (PE), originating from the coatings applied during boxboard manufacturing (Supporting Information). The chemical byproducts represent nonstructural components such as extractives, ash, and degradation products formed during DES pretreatment. Boxboard waste shows the highest proportion of chemical byproducts at 60.6%, followed by wood pulp waste at 48.02%, and sugar cane bagasse at 18.68%. These results highlight the effectiveness of the ternary DES ChCl-EG-OA under a pressure environment in enhancing the cellulose content by effectively removing lignin and facilitating the hemicellulose removal process. However, for processed products such as boxboard waste, the pretreatment parameters can be further optimized to maximize the extraction of specific desired chemicals, such as cellulose.

**Figure 5:**
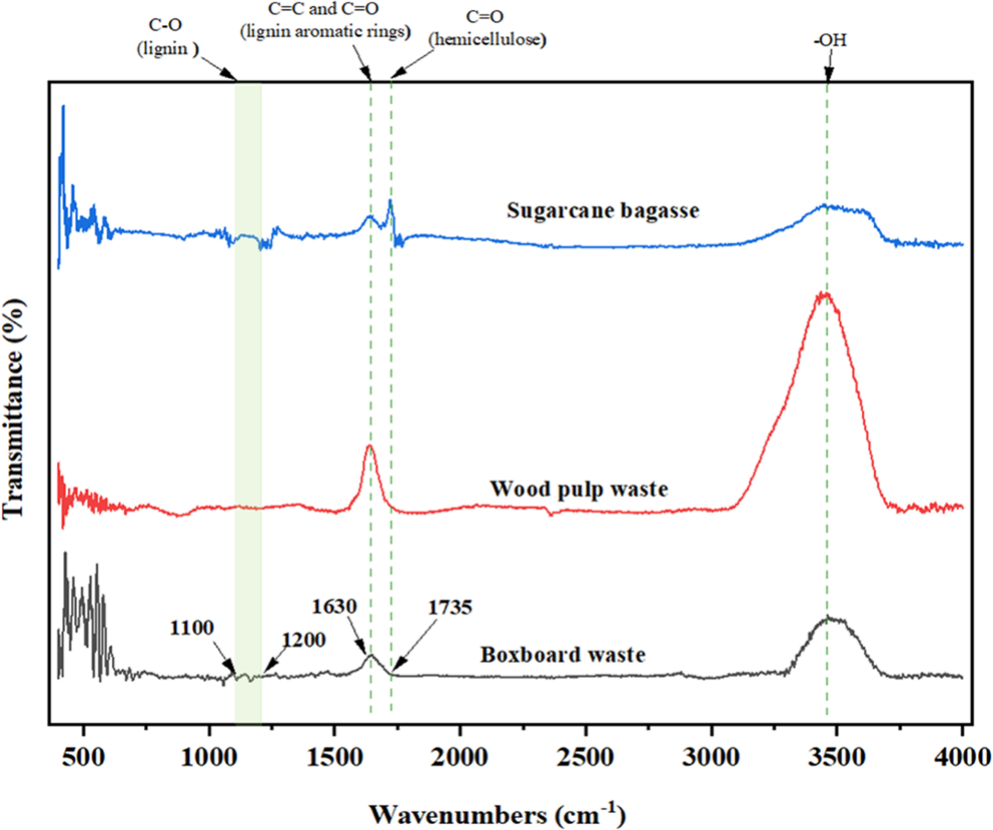
Fourier transform infrared spectra of the cellulose products.

The FTIR spectra, Figure 5, validated the findings of the results. For sugar cane bagasse, the prominent peak at 3400 cm^–1^ (O–H stretching) confirms its cellulose content (72.86%) and the effective removal of hemicellulose and lignin, as indicated by the low intensities at 1735 and 1630 cm^–1^. Wood pulp waste shows significant cellulose peaks at 3400 cm^–1^, consistent with its substantial cellulose content (43.82%), but also retains a high peak at 1630 cm^–1^, indicating residual lignin (8.16%). Additionally, the wood pulp waste sample might contain more residual moisture, which also contributes to the O–H stretching peak. Moisture can increase the intensity of the 3400 cm^–1^ peak, making it appear more pronounced, even with lower cellulose content. Boxboard waste, with clear cellulose peaks at 3400 cm^–1^ and the absence of significant peaks at 1735 cm^–1^ and the band between 1100 and 1200 cm^–1^, confirms the complete removal of hemicellulose and lignin, though it has a lower cellulose content (38.81%).^31^

These FTIR results corroborate the quantitative data, demonstrating the pretreatment method’s efficiency in selectively isolating cellulose, particularly from sugar cane bagasse, and its potential for optimizing cellulose extraction from various biomass sources.

### 3.2 Cellulose Acetate Production

The process of acetylation takes place in two distinct steps. In the initial phase, cellulose fibers (5 g) undergo swelling when exposed to sulfuric acid (0.2 mL) and acetic acid (20 mL) analyzed at a temperature of 60 °C. The swelling process is essential because it enhances the cellulose fibers’ surface area and porosity, enabling better penetration and more efficient reaction of acetic anhydride with the cellulose. Consequently, the enhancement of cellulose pulp activation for acetylation reactions is significantly increased. During the second stage, the acetic anhydride reacts with the hydroxyl groups present on the cellulose fibers’ surface, resulting in the formation of ester linkages and commencing the acetylation reaction. The acetic anhydride permeates the swollen and porous structure of the cellulose fibers, allowing the reaction to proceed more extensively and assuring a comprehensive acetylation process. During this procedure, close attention was paid to the reaction time and the amount of acetic anhydride used in order to achieve the highest possible yield of cellulose acetate. The experiment revealed that using a high-pressure bottle enhances the process’ effectiveness. Using high pressure makes it easier for the reactants to get into the cellulose fibers, which speeds up the reaction and ensures that the acetylation is complete.

**Figure 6:**
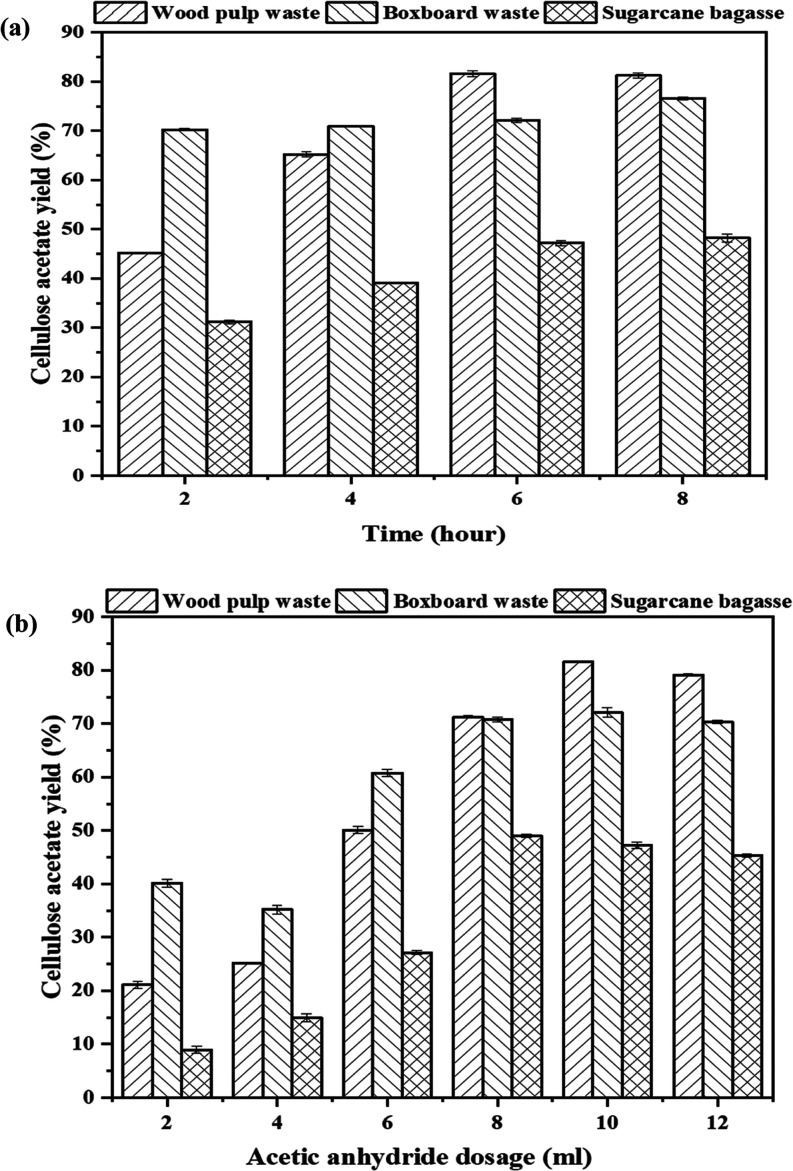
Impact of (a) reaction time and (b) acetic anhydride on cellulose acetate production.

The results in Figure 6 demonstrate clear correlations between the yield of cellulose acetate and both the reaction time and the acetic anhydride dosage for each biomass source. The yield of wood pulp waste increases significantly from 45.23% after 2 h to 81.56% after 6 h. It stabilizes around 81.25% after 8 h. Similarly, the yield rises from 21.1% at 2 mL to 79.13% at 12 mL, reaching the highest at 10 mL (81.56%). Notable increases are observed at 6 mL (50.12%) and 8 mL (71.23%). These results indicate that longer reaction times and higher reagent dosages are necessary for optimal production. The initial yield of boxboard waste is high, starting at 70.26% after 2 h and reaching a peak of 76.56% after 8 h. The yields increase from 40.15% at 2 mL to 70.78% at 8 mL and keep stable to 12 mL (70.36%), with notable increases between 4 mL (35.17%) and 8 mL (70.78%). This suggests that efficient yield can be achieved with moderate amounts of reagent due to the lower lignin content and high initial cellulose. On the other hand, sugar cane bagasse exhibits a gradual increase in yield, starting at 31.17% after 2 h and reaching 48.23% after 8 h. The greatest yield of 47.2% is achieved around the 6 h mark. Furthermore, the yield increases from 8.91% at a dose of 2 mL to 49.01% at 8 mL, and then slightly decreases to 45.32% at 12 mL. This suggests that the best dosage lies around 8 mL. This indicates that achieving the highest possible yield from sugar cane bagasse, which contains hemicellulose and lignin in its complex structure, requires careful control of both reaction time and the amount of acetic anhydride used.

**Figure 7:**
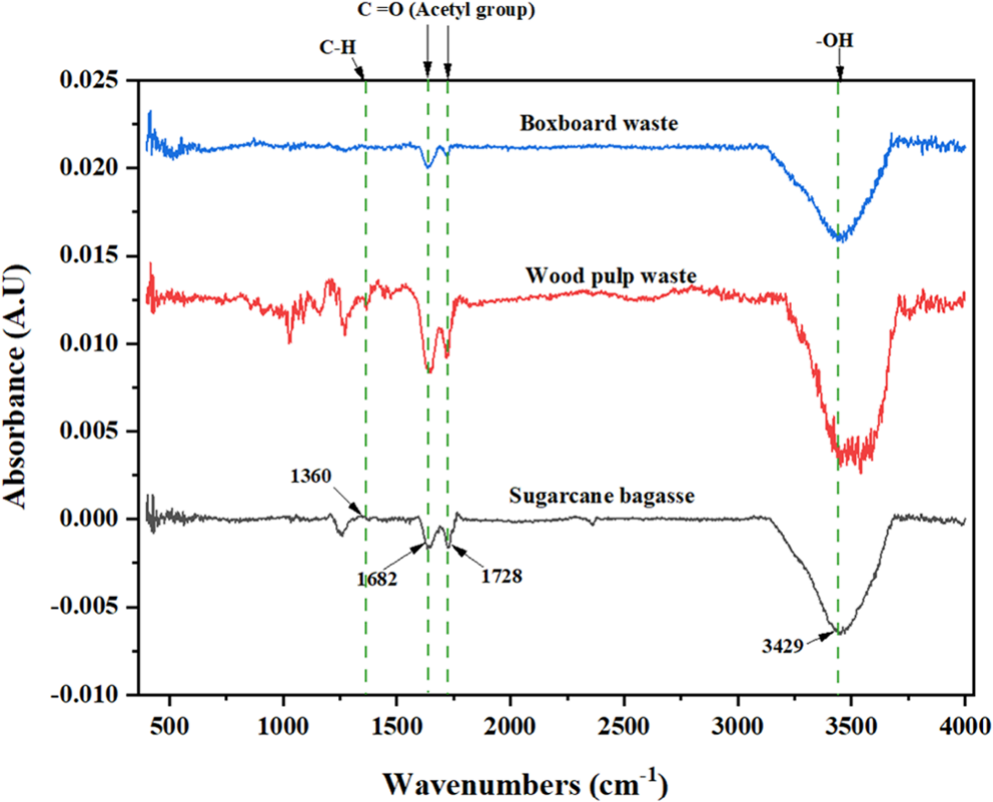
Fourier transform infrared spectra of cellulose acetate products.

Based on the FTIR spectrum shown in Figure 7, the cellulose acetate generated still contains the O–H functional group, which exhibits an absorption peak at 3593 cm^–1^. This suggests that there may be some OH groups that have not been substituted with acetyl groups potentially because the drying process was not fully completed, resulting in the presence of air in the material. The identification of carbonyl groups at the 1682 cm^–1^ band of C=O indicates that cellulose acetate maintains a state of stability. The absorption peak observed at 1728 cm^–1^ is attributed to the oscillation of the C=O bond originating from the carbonyl ester group. Additionally, the increase observed at 1360 cm^–1^ is linked to the vibration of the C–H bond.^32^ When compared with native cellulose, the FTIR spectrum of cellulose acetate shows distinct changes. The characteristic broad O–H stretching peak of cellulose (around 3300–3500 cm^–1^) is significantly reduced in intensity, reflecting the substitution of hydroxyl groups during acetylation. Moreover, the emergence of the carbonyl ester peaks at 1728 and 1360 cm^–1^ in cellulose acetate, absent in native cellulose, highlights the successful incorporation of acetyl groups. These spectral differences provide strong evidence of effective acetylation and validate the chemical modification process.

**Figure 8:**
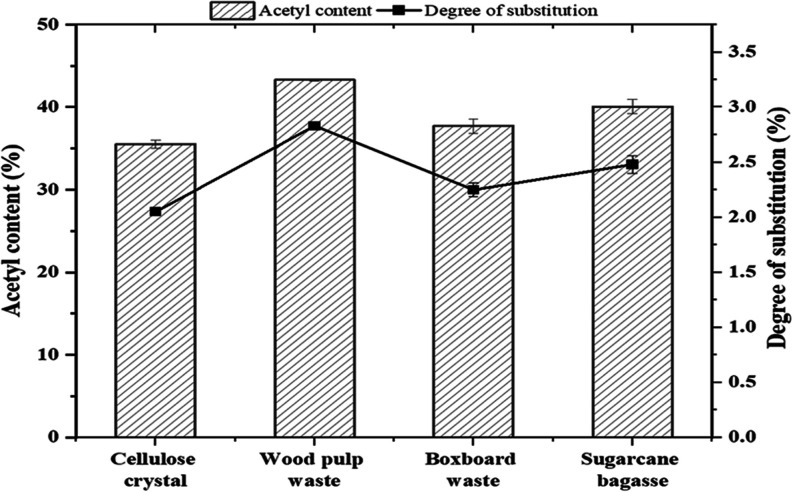
Acetyl content and degree of substitution of various cellulose acetate products.

The degree of substitution (DS) value indicates the average substitution rate of each glucose unit in the polysaccharide.^23^ If all three hydroxyl groups at the C2, C3, and C6 positions of each glucose unit are fully substituted, the DS value reaches its theoretical maximum of 3. A higher DS value is associated with enhanced plastic properties of cellulose esters, as the substitution improves the material’s hydrophobicity and mechanical flexibility.^33^ During the acetylation process, the substitution of hydroxyl groups occurs via an addition–elimination mechanism, where acetic anhydride reacts with the hydroxyl groups to form ester linkages. The −OH group at the C6 position is more reactive and is acetylated more rapidly than the −OH groups at the C2 and C3 positions due to steric hindrances. Among the −OH groups at C2 and C3, the −OH group at C2 is more reactive because it is closer to the hemiacetal structure and is more acidic than the −OH group at C3.^34^ The DS value of cellulose acetate can be accurately measured by using a titration method specific to esters. Based on this method, cellulose acetate was synthesized from cellulose crystal, wood pulp waste, boxboard waste, and sugar cane bagasse used in this study as displayed in Figure 8.

The DS and acetyl content of cellulose acetate derived from various sources reveals significant differences, as displayed in Figure 8, with the lowest DS surprisingly coming from the cellulose crystal. Despite its pure form, cellulose crystal exhibited a DS of 2.05 and an acetyl content of 35.52%, which is unexpected given its typically high reactivity. This lower DS could be attributed to the highly ordered and crystalline structure of cellulose crystals, which may limit the accessibility of hydroxyl groups to the acetic anhydride during the acetylation process. In contrast, wood pulp waste, with the highest acetyl content at 43.32% and a DS of 2.83, indicates extensive acetylation. Boxboard waste and sugar cane bagasse, with acetyl contents of 37.71 and 40.06%, and DS values of 2.25 and 2.48 respectively, fall in between. Wood pulp waste, being more homogeneous and less lignin-rich, allows for more effective acetylation compared to the more complex structures of boxboard waste and sugar cane bagasse.

### 3.3 Bioplastic Film Production

#### 3.3.1 Appearance and Mechanicals of Bioplastic Film

To demonstrate the effectiveness of cellulose acetate synthesis in this study, plastic films were produced from cellulose acetate derived from sugar cane bagasse, boxboard waste, and wood pulp waste. The synthesis process focused on achieving a homogeneous mixture of cellulose acetate without the addition of any other chemicals. Specifically, 0.25 g of cellulose acetate was dissolved in 3 mL of chloroform, and the mixture was stirred for 60 min to ensure thorough dissolution. This method allowed for the evaluation of the resulting plastic films’ mechanical and biodegradability properties, providing insights into the potential applications of cellulose acetate derived from various waste sources. Their appearances are displayed in Figure S1.

The plastic films produced from cellulose acetate derived from different waste sources have different appearances, with the films from sugar cane bagasse and wood pulp waste being more transparent but exhibiting inferior quality, being prone to breaking easily. The boxboard waste-based film, although more yellow in color, stands out due to its superior resilience and durability. This improved performance is likely due to the presence of a polyethylene residue in the cellulose acetate derived from boxboard waste. Polyethylene, originating from coatings in the boxboard, acted as a reinforcing and plasticizing agent, enhancing the molecular interactions within the material. To enhance the properties of the less durable films, cellulose acetate from wood pulp waste was mixed with that from boxboard waste, resulting in a clear, flexible, and promising plastic film.

**Figure 9:**
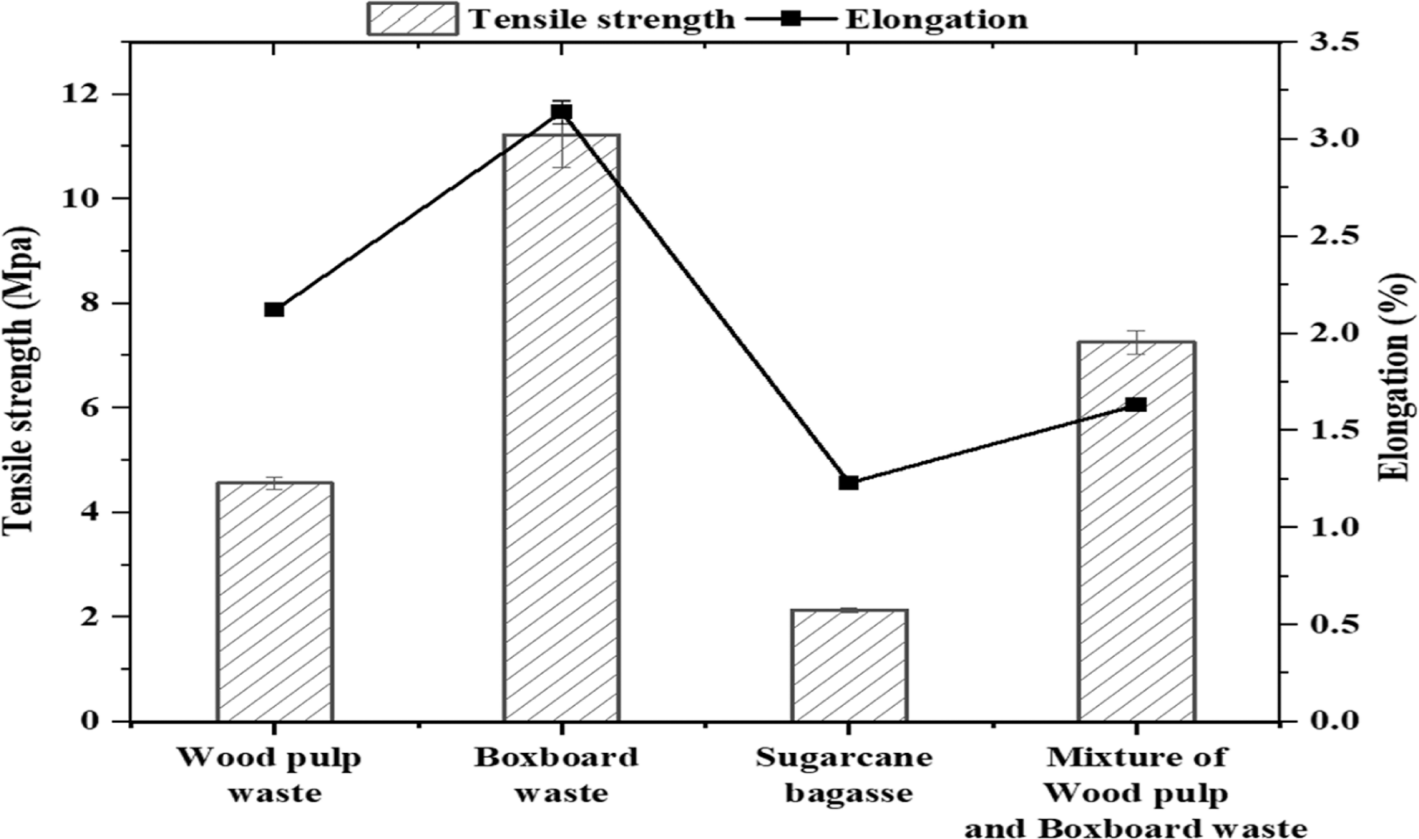
Mechanicals of the bioplastic films.

Figure 9 illustrates the tensile strength and elongation properties of plastics derived from wood pulp waste, boxboard waste, sugar cane bagasse, and a mixture of wood pulp and boxboard waste.

Boxboard waste plastic exhibits the highest tensile strength (11.23 MPa) and elongation (3.14%), making it the most robust and flexible material, likely due to the inherent properties of boxboard fibers and coating chemical enhancements, enhancing the interaction within the cellulose acetate matrix and contributing to improved mechanical properties and greater flexibility. The mixed plastic shows a balanced performance with good tensile strength (around 7.26 MPa) and moderate elongation (approximately 1.63%), suggesting versatility. In contrast, wood pulp waste plastic has a moderate tensile strength (4.56 MPa) and low elongation (slightly above 1.0%), while sugar cane bagasse plastic demonstrates the lowest tensile strength (just above 3.0 MPa) and elongation (1.23%), indicating it is the weakest and least flexible.

#### 3.3.2 Moisture Content, Water Absorption, and Biodegradability of Plastic Film

Table 6 presents data on the moisture content and water absorption of plastics derived from sugar cane bagasse, boxboard waste, wood pulp waste, and a mixture of these materials. The sugar cane bagasse and wood pulp waste plastics show lower moisture content (1.21 and 1.02%, respectively) and water absorption (0.75 and 0.57%, respectively), indicating better inherent water resistance and potential durability. In contrast, boxboard waste plastic, despite having a higher moisture content (1.79%) and water absorption (1.95%), exhibits better structural integrity. The mixed plastic, combining cellulose acetate from boxboard waste and wood pulp waste, shows moderate values for the moisture content (1.46%) and water absorption (0.91%), suggesting a balanced performance. This mixed-source plastic leverages the strengths of each individual material, resulting in improved overall properties, and making it a promising candidate for applications requiring both durability and water resistance. This approach underscores the potential of combining different cellulose acetate sources to produce versatile and biodegradable plastics that are suitable for diverse uses.

**Table 6:** Moisture Content and Water Absorption Measurement

plastic types	moisture content, %	water absorption, %
sugar cane bagasse-based plastic	1.21	0.75
boxboard waster-based plastic	1.79	1.95
wood pulp waste plastic	1.02	0.57
mixture of boxboard and wood pulp plastic	1.46	0.91

The degradability of cellulose acetate plastics was evaluated over a period of 5 days and reveals a distinct order in this study.

Table 7 reveals that the biodegradability of plastics derived from boxboard waste-based plastic exhibits the highest weight loss (13.03%), indicating the fastest degradation, followed by the mixture plastic (9.73%), sugar cane bagasse-based plastic (9.50%), and wood pulp waste plastic (5.52%). Compared to other studies, these plastics degrade faster in the short term than polylactic acid (20.00% over 20 days) but less extensively than polyhydroxyalkanoates (PHA) (89.75% over 30 days), potato-based bioplastic (43.00% over 7 days), and polyhydroxyurethanes hybrid (88.00% over 121 days). The mixed-source plastic offers balanced biodegradability, making it a versatile option for applications requiring moderate degradation rates. These comparisons highlight the potential of using different cellulose acetate sources to produce biodegradable plastics with desirable degradation rates for various applications.

**Table 7:** Comparison of Cellulose Acetate Plastic to Various Renewable Plastics

		biodegradability		
plastic type	env.	weight loss, %	time, days	references
sugar cane bagasse-based plastic	soil	9.50	5	this study
boxboard waster-based plastic	soil	13.03	5	this study
wood pulp waste plastic	soil	5.52	5	this study
mixture of boxboard and wood pulp plastic	soil	9.73	5	this study
polylactic acid	soil	20.00	20	(35)
polyhydroxyalkanoates	soil	89.75	30	(36)
potato-based bioplastic	soil	43.00	07	(37)
polyhroxyurethanes hybrid	soil	88.00	121	(38)
blended PLA/polybutylene adipate (PBAT)	soil	60.16	105	(38)

## 4 Conclusions

This study thoroughly explores the production of bioplastic films from sugar cane bagasse, wood pulp waste, and boxboard waste through a three-step process. Initially, cellulose was extracted from biomass using a ternary deep eutectic solvent (DES) system—ChCl-EG-OA. The findings highlight that DES pretreatment significantly enhances lignin removal and facilitates a more effective alkaline process for hemicellulose removal. Under optimal conditions, the extraction efficiency for sugar cane bagasse resulted in the highest cellulose content at 72.86%, with 3.45% hemicellulose and 5.01% lignin. Wood pulp waste showed a substantial cellulose content of 43.82% but also contained 8.16% lignin. Boxboard waste, although free of hemicellulose and lignin post-pretreatment, had a lower cellulose content at 38.81%. However, its unique composition, including residual polyethylene and chemical byproducts, provides its potential for further exploration for bioplastic production and other value-added applications and extractions, which were not fully addressed in this study.

In the second phase, various reaction conditions were tested to determine the optimal method for cellulose acetate production. The results indicated that wood pulp waste achieved the highest yield at around 81.25%, followed by boxboard waste at 70.78% and sugar cane bagasse at the lowest yield of 47.2%. Furthermore, wood pulp waste had the highest acetyl content, 43.32%, with a degree of substitution (DS) of 2.83, indicating extensive acetylation. Boxboard waste and sugar cane bagasse had acetyl contents of 37.71 and 40.06%, with DS values of 2.25 and 2.48, respectively. The characteristics of the bioplastic films varied significantly depending on the biomass source of cellulose acetate. Boxboard waste-derived plastic exhibited the highest tensile strength (11.23 MPa) and elongation (3.14%), likely due to its fiber composition and possible chemical enhancements. Wood pulp waste-derived plastic had moderate tensile strength (4.56 MPa) and low elongation (just above 1.0%), while sugar cane bagasse-derived plastic was the weakest and least flexible among the samples. The mixed-source plastic between boxboard waste wood pulp showed enhanced strong tensile strength (7.26 MPa) and moderate elongation (1.63%), highlighting their potential for diverse packaging uses.

Finally, this study serves as a fundamental reference for the exploration of waste utilization and demonstrates the potential of combining the advantages of different biomass sources for the production of new, sustainable materials without the addition of toxic chemicals.

## Supporting Information Available

Characterization of biomass (Table S1); components of synthesized DESs (Table S2); appearance of bioplastic films (Figure S1); and method for polyethylene isolation and the FTIR result (Figure S2) (PDF)
