# Supplementary material for: Sustainable Production of High-Performance Bioplastics from Agricultural and Industrial Biomass Waste by Integrating Deep Eutectic Solvent (DES) Pretreatment and Acetylation Processes
Source: ACS Omega. 2025 Mar 11;10(11):10949–61. doi: 10.1021/acsomega.4c09059 (PMC11947807; doi:10.1021/acsomega.4c09059)
Supplement: Supplementary file 1 — ao4c09059_si_001.pdf [file ao4c09059_si_001.pdf]

Supporting Information

Sustainable Production of High-Performance Bioplastics from Agricultural and Industrial Biomass Waste by Integrating Deep Eutectic Solvent (DES) Pretreatment and Acetylation Processes

Natcha Chyerochana[1], Quang Tam Huynh[2], Udomsap Jaitham[3], Paripok Phitsuwan[1], Kornkanok Aryusuk[1], Surat Hongsibsong[3, 4], Ku-Fan Chen[5], Ken-Lin Chang[2, 6, 7, 8, *]

[1]Division of Biochemical Technology, School of Bioresources and Technology, King Mongkut's University of Technology Thonburi, Bangkuntien, Bangkok, Thailand.

[2]Institute of Environmental Engineering, National Sun Yat-Sen University, Kaohsiung 804, Taiwan.

[3]School of Health Sciences Research, Research Institute for Health Sciences, Chiang Mai University, Chiang Mai 50200, Thailand.

[4]Environment, Occupational Health Sciences and Non-Communicable Disease Center of Excellence, Re-search Institute for Health Sciences, Chiang Mai University, Chiang Mai 50200, Thailand.

[5]Department of Civil Engineering, National Chi Nan University, Nantou 545, Taiwan

[6]Net Zero Emissions and Resource Recycling Technology Research Center, National Sun Yat-Sen University, Kaohsiung 804, Taiwan.

[7]Department of Public Health, College of Health Sciences, Kaohsiung Medical University, Kaohsiung 807, Taiwan.

[8]Center for Emerging Contaminants Research, National Sun Yat-Sen University, Kaohsiung 804, Taiwan.

N.C. and Q.T.H. contributed equally in this work and should be designated as co-first authors.

*Corresponding author at: Institute of Environmental Engineering, National Sun Yat-Sen University, Kaohsiung 804, Taiwan.

E-mail address: klchang@mail.nsysu.edu.tw *(K.-L. Chang)*.

1. Table S1. The characterization of biomass

Parameters (%)	Sugarcane bagasse	Boxboard waste	Wood pulp waste
- Cellulose	37.77 ± 0.38%	41.81 ± 0.12%	43.82 ± 0.77%
- Hemicellulose	24.22 ± 1.08%	5.20 ± 0.07%	3.39 ± 0.19%
- Lignin	23.70 ± 0.40%	13.38 ± 1.32%	41.35 ± 0.55%
- Ash	4.53± 0.35%	11.06±1.09%	0.26±0.08%
- Others	9.78±2.21%	26.55±2.6%	11.18±1.59%

2. Table S2: The components of synthesized DESs

Entry	HBA	HBD-1	HBD-2	Molar Ratio	DESs abbreviations
1	Choline Chloride	Ethylene glycol	Oxalic acid	1:2:0.6	ChCl-EG-OA
2	Choline Chloride	Ethylene glycol	Formic acid	1:2:0.6	ChCl-EG-FA
3	Choline Chloride	Ethylene glycol	Malonic acid	1:2:0.6	ChCl-EG-MA

3. Appearance bioplastic films

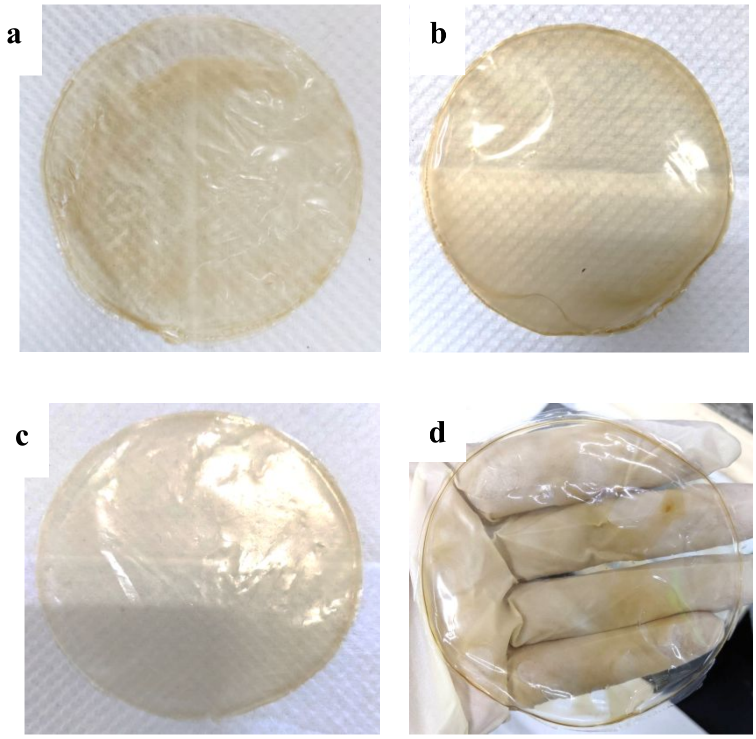

Figure S1. Appearance bioplastic films, (a)- sugarcane bagasse, (b)-boxboard waste, (c)- wood pulp waste, (d)- wood pulp waste and boxboard waste mixture.

4. Polyethylene isolation

The process was modified form the study by Wong and colleagues. [1] Polyethylene was isolated by treating 1 g of cellulose of raw boxboard waste separately with p-xylene (1 g:10 mL) at 130 °C for 24 hours under stirring. After dissolving polyethylene, the mixture was hot-filtered to separate the undissolved cellulose. The filtrate was cooled,

and acetone was added to precipitate polyethylene, which was collected by centrifugation, washed with acetone, and dried at 60 °C to constant weight. The amount of product was quantified gravimetrically, yielding 0.0059 g of polyethylene.

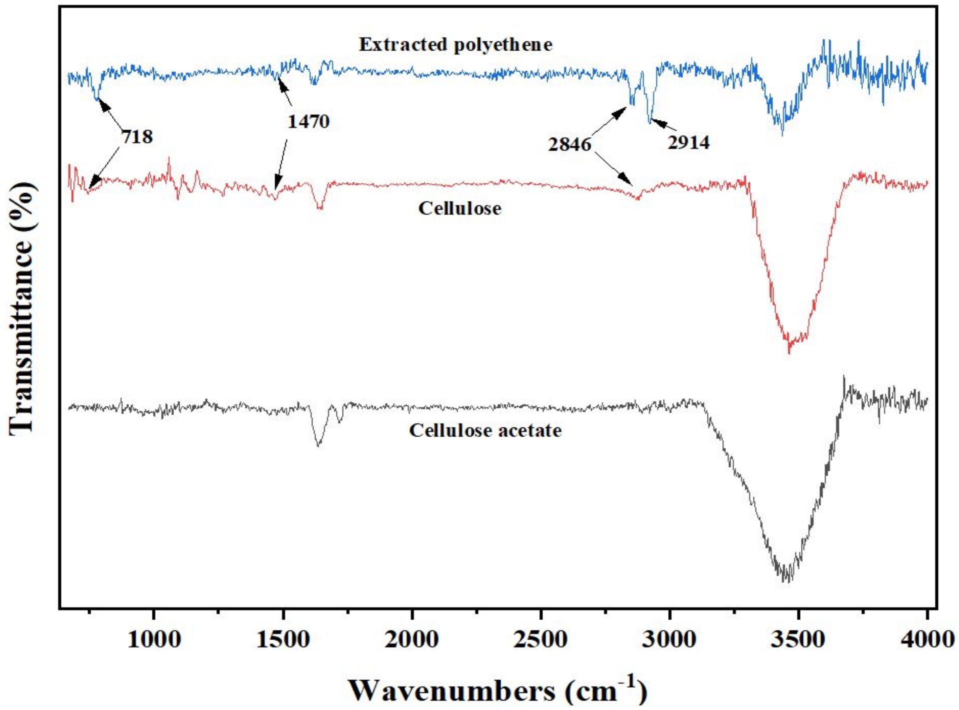

Figure S2. FTIR spectrum of cellulose and cellulose acetate derived from boxboard waste.

The spectrum of extracted polyethylene exhibits characteristic peaks at 2914 cm^{-1}, 2846 cm^{-1}, and 1470 cm^{-1}, [2] corresponding to the asymmetric and symmetric stretching of CH_2 groups, respectively, as well as a peak at 718 cm^{-1}, attributed to CH_2 rocking vibrations. [3] These peaks confirm the successful isolation of polyethylene from the boxboard waste.

This FTIR spectrum serves as a valuable demonstration of the presence of polyethylene in the boxboard-derived cellulose. The characteristic peaks at 2846 cm^{-1}, 1470 cm^{-1}, and at 718 cm^{-1} is confirmed the presence of PE. Compared to cellulose, the cellulose acetate spectrum preserves these distinctive PE peaks, albeit to a lesser extent, suggesting that the polyethylene residue remains integrated even after the acetylation process. This residual polyethylene contributes to the enhanced mechanical properties, such as the increased tensile strength and elongation, by reinforcing the cellulose acetate matrix.

References

(1) Wong, P. K.; Lui, Y. W.; Tao, Q.; Lui, M. Y. Solvent-targeted recovery of all major materials in beverage carton packaging waste. *Resources, Conservation and Recycling* **2024**, *202*, 107367. DOI: https://doi.org/10.1016/j.resconrec.2023.107367.

(2) D'Amelia, R.; Gentile, S.; Nirode, W.; Huang, L. Quantitative Analysis of Copolymers and Blends of Polyvinyl Acetate (PVAc) Using Fourier Transform Infrared Spectroscopy (FTIR) and Elemental Analysis (EA). *World Journal of Chemical Education* **2016**, *4*, 25-31. DOI: 10.12691/wjce-4-2-1.

(3) Anju Abraham, P.; Gokul, V.; Swapna, M. N. S.; Sankararaman, S. I. Thermal lens technique's surrogacy unveiled: A novel tool for microplastic detection and quantification in water. *Heliyon* **2024**, *10* (14), e34532. DOI: https://doi.org/10.1016/j.heliyon.2024.e34532.
